# International Medical Congress

**Published:** 1881-07

**Authors:** J. Risdon Bennett, William Mac Cormac

**Affiliations:** Chairman of the Executive Committee; Secretary-General


### INTERNATIONAL MEDICAL CONGRESS.

### SEVENTH SESSION.

At the last meeting of this body held last September in Amsterdam, it was decided to hold the meeting in August, 1881, in Great Britain, provided the arrangement was acceptable to the medical profession of that Country. The approval was most cordially given, and a hearty invitation extended to the medical profession of the world to be present. The occasion will be one of great interest. In evidence of this fact, the following from the general circular is given, viz.: "Her Majesty the Queen has most graciously given proof of her good will towards the cause of Medical Science, and our efforts in its furtherance, by authorizing us to place the Congress under Her Royal patronage.

His Royal Highness the Prince of Wales has likewise shown the unvarying interest he takes in the progress of Medicine by according a similar favor.

The work of the Congress will be carried on in fifteen Sections. The days of the meeting will extend from Wednesday, the 3rd, to Tuesday, the 9th of August, both days included. A Reception of Welcome will take place on the evening of August 2nd.

The meetings will be chiefly held in the Halls of the University of London, and in Burlington House, where in a most liberal manner the use of rooms for the General and Sectional Meetings has been granted to the Congress by the authorities of the University of London, the Royal Society, the Society of Antiquaries, the Astronomical Society, the Linnean Society, the Chemical Society, and the Geological Society.

There will be a Museum open during the Meeting, to which contributions of professional interest will be made. Evening Receptions will be held, and Excursions arranged to various places of interest.

The attendance of our countrymen from all parts of the United Kingdom, India, and the Colonies, will probably be large, and various circumstances make it probable that a large number of distinguished men from many countries will be attracted to England as our guests on the occasion of the Seventh Session of the Congress, and it is our desire to receive them with all cordiality and honor.

It is convenient to inform our colleagues abroad that ladies will be invited to the Social and Ceremonial Meeting of the Congress, but will not be admitted to its Business Meetings.

It will be necessary for all who wish to make communications to the Congress to intimate their intentions to the Secretaries of the several Sections, after which a Programme of the Business will be issued.

J. RISDON BENNETT,
*Chairman of the Executive Committee.*
WILLIAM MAC CORMAC,
*Secretary-General.*

All communications respecting the Congress should be adressed to William Mac Cormac, Esq., Hon. Secretary-General,
13, Harley Street, London, W.

